# En bloc resection and trapezius island flap reconstruction of giant extracranial Schobinger stage III/Yakes type IV arteriovenous malformations involving the orbital region

**DOI:** 10.1016/j.jpra.2026.08.021

**Published:** 2026-08-21

**Authors:** Wei-liang Chen, Bin Zhou, Zi-xian Huang, Hai-gang Li, Rui Chen, You-yuan Wang

**Affiliations:** a Professor and Director, Department of Oral and Maxillofacial Surgery, Sun Yat-sen Memorial Hospital, Sun Yat-sen University, 107 Yan-jiang Road, Guangzhou, 510120, China; b Professor, Department of Oral and Maxillofacial Surgery, Sun Yat-sen Memorial Hospital, Sun Yat-sen University, Guangzhou, 510120, China; c Attending, Department of Oral and Maxillofacial Surgery, Sun Yat-sen Memorial Hospital, Sun Yat-sen University, Guangzhou, 510120, China; d Professor and Director, Department of Pathology, Sun Yat-sen Memorial Hospital, Sun Yat-sen University, Guangzhou, 510120, China

**Keywords:** Arteriovenous malformation, Congenital vascular anomaly, Head and neck, Cranio-maxillo-facial defects, Surgical management, En bloc resection, Trapezius flap

## Abstract

**Background:**

Extensive head and neck arteriovenous malformations (AVMs) associated with severe hemorrhage are potentially life-threatening and present substantial challenges for head and neck surgeons.

**Methods:**

We evaluated a 33-year-old man (Patient 1) and a 38-year-old woman (Patient 2) with life-threatening, extensive Schobinger stage III/Yakes type IV AVMs of the head and neck. Both patients underwent en bloc resection, and the resulting large craniofacial defects were reconstructed using an extended vertical trapezius myocutaneous island flap (eTMF). In Patient 2, simultaneous radical neck dissection was performed because of a concomitant primary T4a squamous cell carcinoma of the maxilla.

**Results:**

The extensive head and neck AVMs, including those with orbital involvement, were completely and safely resected, and the foldable eTMF reconstructed the resulting large defects. No surgical complications occurred, and postoperative appearance was acceptable. Swallowing, mastication, and speech were restored to preoperative levels. Quality-of-life scores improved by 40 percentage points, reaching 80% in Patient 1 and 60% in Patient 2. Patient 1 remained disease-free after 36 months of follow-up, whereas Patient 2 died of local recurrence 8 months after surgery.

**Conclusions:**

En bloc resection or radical surgery is an effective treatment approach for patients with life-threatening extracranial Schobinger stage III/Yakes type IV AVMs involving the orbital region, even when accompanied by T4a-stage SCC of the maxilla. The foldable eTMF is a large, highly reliable option for reconstruction of extensive head and neck defects, including full-thickness cheek defects.

## Introduction

Arteriovenous malformations (AVMs) are rare congenital vascular anomalies characterized by direct communication between arteries and veins that bypass the capillary bed or by the absence of a normal capillary network. Extracranial AVMs most commonly involve the head and neck region (47.4%), followed by the extremities (28.5%).^1^^,^^2^ Clinical manifestations are heterogeneous, ranging from asymptomatic cutaneous lesions present at birth to pain, bleeding, craniofacial deformities, functional impairment, and life-threatening massive hemorrhage. According to the Schobinger staging system, clinical manifestations progress from stage I (quiescence), characterized by cutaneous blush and warmth; to stage II (expansion), characterized by lesion enlargement, bruit, and palpable or audible pulsations; to stage III (destruction), characterized by pain, ulceration, bleeding, and infection; and finally to stage IV (decompensation), characterized by cardiac failure.^3^ Angiographically, AVMs are classified according to the Yakes classification into six types: type I, direct arteriovenous fistula; type IIA, nidus-type AVM; type IIB, nidus-type AVM with aneurysmal venous drainage; type IIIA, multiple arterial inflows with a single aneurysmal venous outflow; type IIIB, multiple arterial feeders with multiple venous outflows and an aneurysmal venous pouch; and type IV, innumerable arteriovenous connections at the arteriolar level.^4^

Patients with Schobinger stage I and II lesions, particularly pediatric patients, are recommended to undergo annual assessments, with intervention indicated if progression to stage III occurs.^5^ In a study of endovascular treatment for head and neck AVMs using ethanol and liquid embolic agents, the Schobinger stage was reduced in all patients. The elimination rate for arteriovenous shunts exceeded 90% in most cases. Lesions classified as Yakes type IV required more treatment sessions than those in the type II and type III groups.^6^ Stage III oral and maxillofacial AVMs are managed using a multidisciplinary approach. Treatment modalities include superselective intra-arterial embolization, sclerotherapy, bone wax packing of osseous cavities with curettage, and surgical resection, applied alone or in combination.^7^, ^8^, ^9^ A multidisciplinary strategy also encompasses defect planning and reconstruction, including the use of free radial forearm flaps, anterolateral thigh flaps, and vascularized fibular flaps.^10^ Successful surgical management, with or without adjunctive embolization, requires complete removal of the nidus. Large, focal, or diffuse lesions involving multiple anatomic regions typically necessitate surgical reconstruction.^11^

These lesions may be life-threatening because of progressive symptoms and their infiltrative nature. However, surgical management of large and complex AVMs is particularly challenging, as complete lesion excision, effective hemorrhage control, and simultaneous defect reconstruction are difficult to achieve. We present two cases of life-threatening giant extracranial Schobinger stage III/Yakes type IV AVMs involving the orbital region that were treated successfully with en bloc resection and subsequent reconstruction using an extended vertical trapezius myocutaneous island flap (eTMF).

## Materials and methods

Two patients with extensive AVMs underwent en bloc resection with defect reconstruction using an eTMF in August 2020 and May 2023. The institutional review board and the ethics committee approved the study protocol. Diagnosis was established on the basis of medical history, physical examination, magnetic resonance imaging, computed tomography, computed tomography angiography, and histopathologic findings. Lesions were staged and classified using the Schobinger staging system and the Yakes classification, respectively, based on clinical presentation and angiographic characteristics. Surgical planning was confirmed through a multidisciplinary team (MDT) approach.

At 6 months postoperatively, a panel of three surgeons evaluated the range of shoulder motion at the donor site, including internal and external rotation.^12^ The patients completed the University of Washington Quality of Life Questionnaire (version 4; UW-QOL), which includes domains assessing swallowing, chewing, speech, and overall quality of life.^13^

## Case presentation

### Case 1

**Fig. 1:**
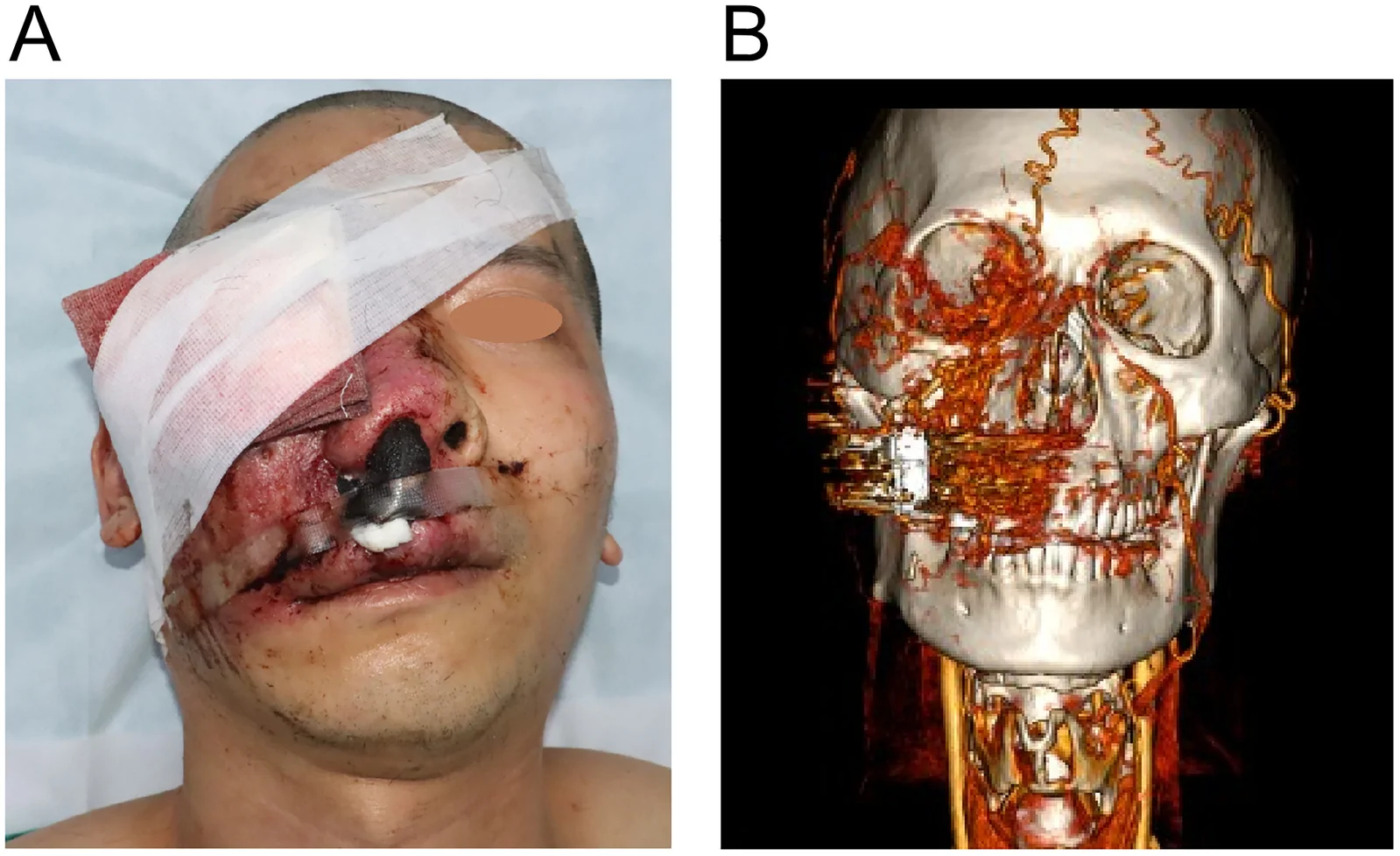
A 33-year-old man with a giant Schobinger stage III/Yakes type IV arteriovenous malformations (AVMs) involving the right orbit and maxillofacial region (Case 1). The patient presented with uncontrolled massive hemorrhage (A). Computed tomography angiography demonstrates that the lesion is supplied predominantly by the right ophthalmic artery arising from the internal carotid artery, with venous drainage into the right internal and external jugular veins (B).

A 33-year-old man was born with a blanching, warm mass in the right maxillofacial region. He presented with sudden, profuse hemorrhage that rapidly became uncontrolled and life-threatening, prompting emergency transfer to our hospital for treatment (Fig. 1). The lesion had been diagnosed as an AVM at 3 years of age (Schobinger stage I) and was initially managed conservatively without intervention. By 21 years of age, the lesion had progressed to stage II, and the patient underwent multiple embolization and sclerotherapy procedures. At 30 years of age, the lesion progressed to stage III. Despite repeated interventions, including superselective intra-arterial embolization, combined compartmentalization and sclerotherapy, and tooth extraction with bone wax packing of the osseous cavity, the disease remained uncontrolled. Histopathologic examination confirmed the diagnosis of a vascular malformation. The lesion was classified as a Schobinger stage III/Yakes type IV AVM. Table 1 presents a detailed summary of the medical history and clinical findings.

**Table 1:** Demographic data and clinical characteristics of two patients with AVMs.

	Patient 1	Patient 2
**Sex/age (years)**	Male/33	Female/38
**Schobinger stage I**	At birth, a blushing mass was noted on the right cheek	In infancy, a blushing mass was noted on the left cheek.
**Schobinger stage II**	At age 3 years, the lesion involved the right orbit and was diagnosed as an AVM; no intervention was performed. At age 21 years, the patient underwent multiple embolization and sclerotherapy procedures, although detailed records were unavailable.	After age 10 years, the AVM progressively enlarged and spread to the craniofacial region. No intervention was performed during this period.
**Schobinger stage III**	Within 3 years after age 30 years, the patient experienced > 10 episodes of right facial AVM–related severe bleeding and underwent repeated SIAEs. Embolic materials included ethylene vinyl alcohol copolymers, polyvinyl alcohol particles, and coils. Two years later, recurrent bleeding occurred, and the patient underwent three sessions of combined compartmentalization and sclerotherapy, extraction of the right upper teeth, and bone wax packing of the bone cavities. Subsequently, massive hemorrhage suddenly occurred from the right canthus and became uncontrollable, prompting emergency transfer to our hospital.	Over the 20 years following age 10 years, the patient experienced recurrent bleeding from the left upper gingiva and left nasal cavity. She underwent multiple AVM SIAEs, multiple intratumoral injections of anhydrous alcohol, and > 10 intratumoral injections of bleomycin. In recent years, the craniofacial mass increased markedly in size and was accompanied by pain and bleeding. She was hospitalized because the lesion had progressed to life-threatening ulcerative hemorrhage.
**Clinical physical examination**	A massive AVM involving the right orbit, nose, cheek, and upper lip, with severe active bleeding from the orbit and nostrils.	A massive AVM involving the left forehead, orbit, nose, cheek, and upper lip. The left globe was markedly protruded. The left nasal ala and upper lip were atrophic and deformed. Multiple nodular masses with ulceration were present in the infraorbital region and cheek.
**Imaging findings**	CT showed an irregular soft-tissue mass in the right maxillofacial region involving the right orbital floor, lacrimal gland, lacrimal sac, pterygopalatine fossa, cheek, and maxilla, measuring approximately 7.5 × 3.5 cm. Dense nodular opacities and multiple metallic densities were present. CTA demonstrated that the lesion was mainly supplied by the right ophthalmic artery arising from the internal carotid artery, with venous drainage into the right internal and external jugular veins (Yakes IV)	MRI demonstrated an ill-defined soft-tissue mass measuring approximately 7.5 × 6.5 × 6.5 cm in the left craniofacial region. The lesion invaded the left maxilla from the infraorbital wall to the anterior walls of the orbit and maxillary sinus, resulting in skin ulceration and cheek defects. Multiple enlarged lymph nodes were observed in left cervical levels I and II. CTA showed that the tumor was supplied by the left maxillary artery, with local vascular disorganization, thickening, and post-treatment changes consistent with a high-flow vascular malformation (Yakes IV).
**Pathological diagnosis**	Vascular malformation involving deep adipose and fibrous tissue within striated muscle	Squamous cell carcinoma associated with vascular malformations

AVM, arteriovenous malformations; SIAE, superselective intra-arterial embolization; MRI, Magnetic resonance imaging; CT, computed tomography; CTA, computed tomography angiography.

At the MDT meeting, both the diagnosis and therapeutic strategy were confirmed. The patient was determined to have a life-threatening Schobinger stage III AVM involving the right orbital and maxillofacial region, resulting in complete loss of vision in the right eye, with no evidence of intracranial extension. Emergency en bloc resection of the AVM, including orbital content removal, was deemed necessary. The planned perioperative strategy to control hemorrhage and maintain hemodynamic stability included preoperative autologous blood donation, ligation of the right external carotid artery prior to tumor resection, incision along normal skin at the tumor margin, and use of the Harmonic ACE+7 surgical scalpel (Ethicon Endo-Surgery, Cincinnati, OH, USA) for lesion excision. Following complete AVM resection, reconstruction of the resulting large defect using eTMF was considered safe and reliable.^14^

### Case 2

**Fig. 2:**
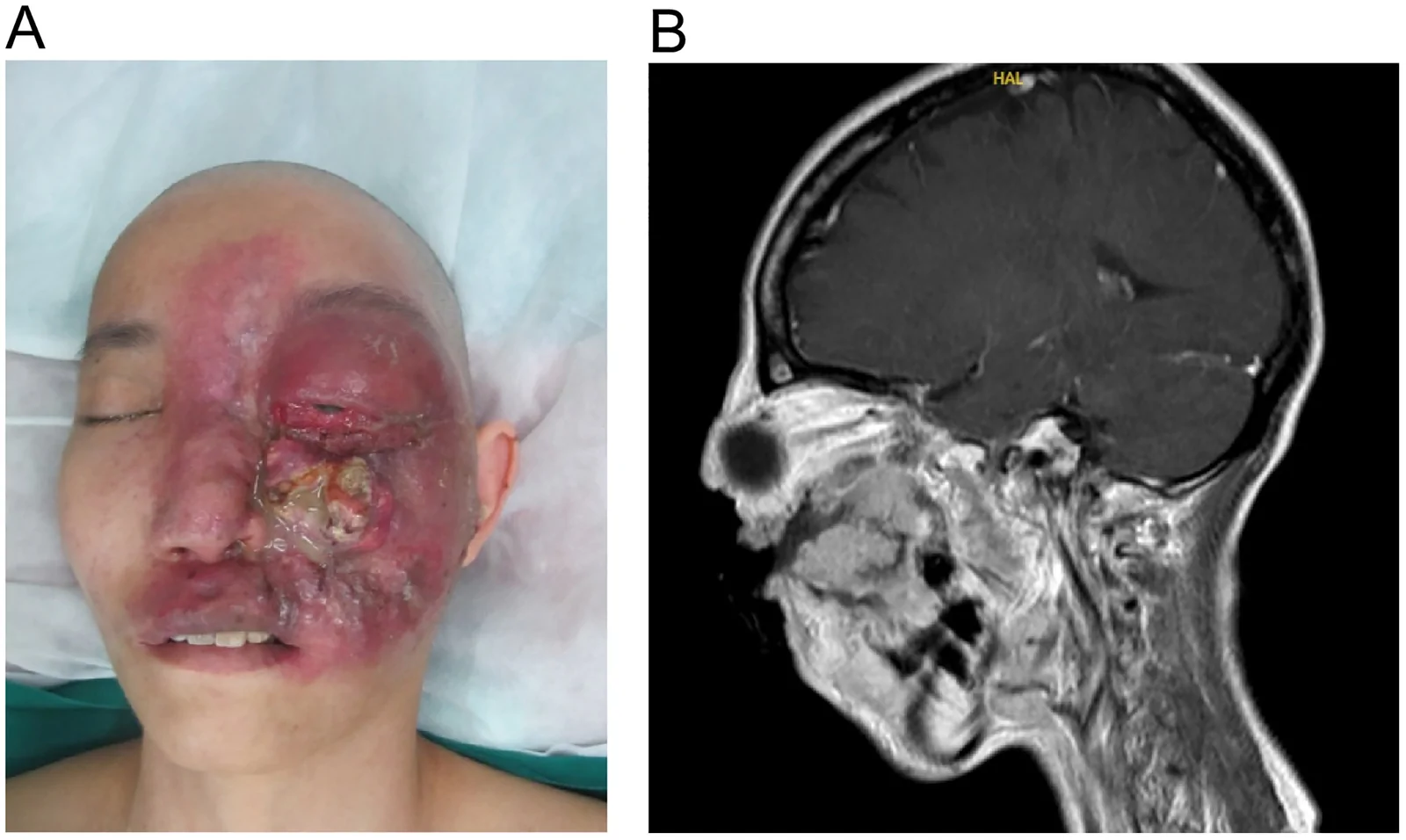
A 38-year-old woman with a Schobinger stage III/Yakes type IV AVMs associated with left maxillary squamous cell carcinoma (Case 2). A large blushing mass occupies the left craniomaxillofacial region, with tumor ulceration and active bleeding (A). Magnetic resonance imaging demonstrates a large soft-tissue mass in the left craniofacial region invading the craniofacial bones and orbit, resulting in cheek skin ulceration and tissue defects (B).

**Fig. 3:**
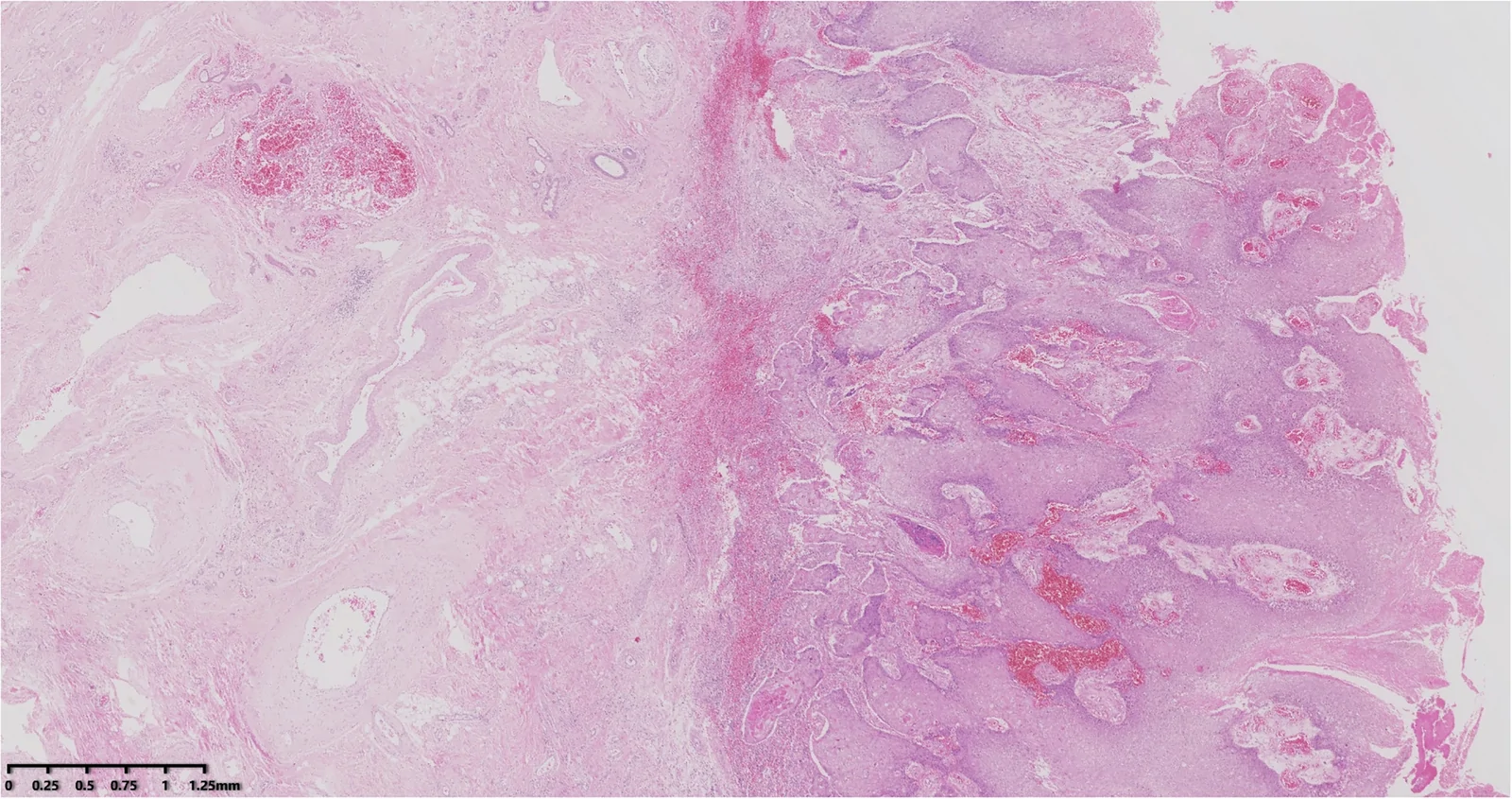
Hematoxylin and eosin staining demonstrates surface squamous cell carcinoma with papillary growth and stromal invasion, with malformed vessels of variable size and irregular morphology observed in the deeper tissue layers.

A 38-year-old unmarried woman was admitted with a left-sided oral and maxillofacial bluish mass that had been present for > 30 years and had developed ulceration and bleeding during the preceding 2 months (Fig. 2). At 10 years of age, the lesion was diagnosed as a stage II AVM. During the subsequent 20 years, the lesion progressed to stage III, with recurrent episodes of significant spontaneous bleeding involving the left upper gingiva and the left nasal cavity. The patient underwent multiple sessions of AVM superselective intra-arterial embolization, multiple anhydrous alcohol injections, and > 10 intratumoral injections of pingyangmycin; however, the lesion remained uncontrolled. In recent years, the craniofacial mass progressively enlarged and was associated with substantial pain and intermittent bleeding. Two months before admission, ulcerative bleeding developed in the left maxillary, orbital, and cheek regions. On evaluation, the AVM was classified as Schobinger stage III/Yakes type IV, with concomitant squamous cell carcinoma (SCC) of the left maxilla (Fig. 3). Table 1 presents a detailed medical history and the results of relevant examinations.

The MDT determined the diagnostic and therapeutic strategy. It was confirmed that the patient had a large Schobinger stage III/Yakes type IV AVM involving the left orbit and maxillofacial region, accompanied by clinically staged T4a SCC of the left maxilla, according to the eighth edition of the American Joint Committee on Cancer oral cancer staging system.^15^ The tumor was actively bleeding, and the patient had complete loss of vision in the left eye. Considering the malignant nature of the disease, definitive management was planned as for an advanced malignancy, including en bloc resection of the orbital contents, extended resection of the maxillary tumor, and radical neck dissection. Following en bloc orbital exenteration and extended maxillary resection, reconstruction of the large through-and-through defect was planned using a folded eTMF.

**Fig. 4:**
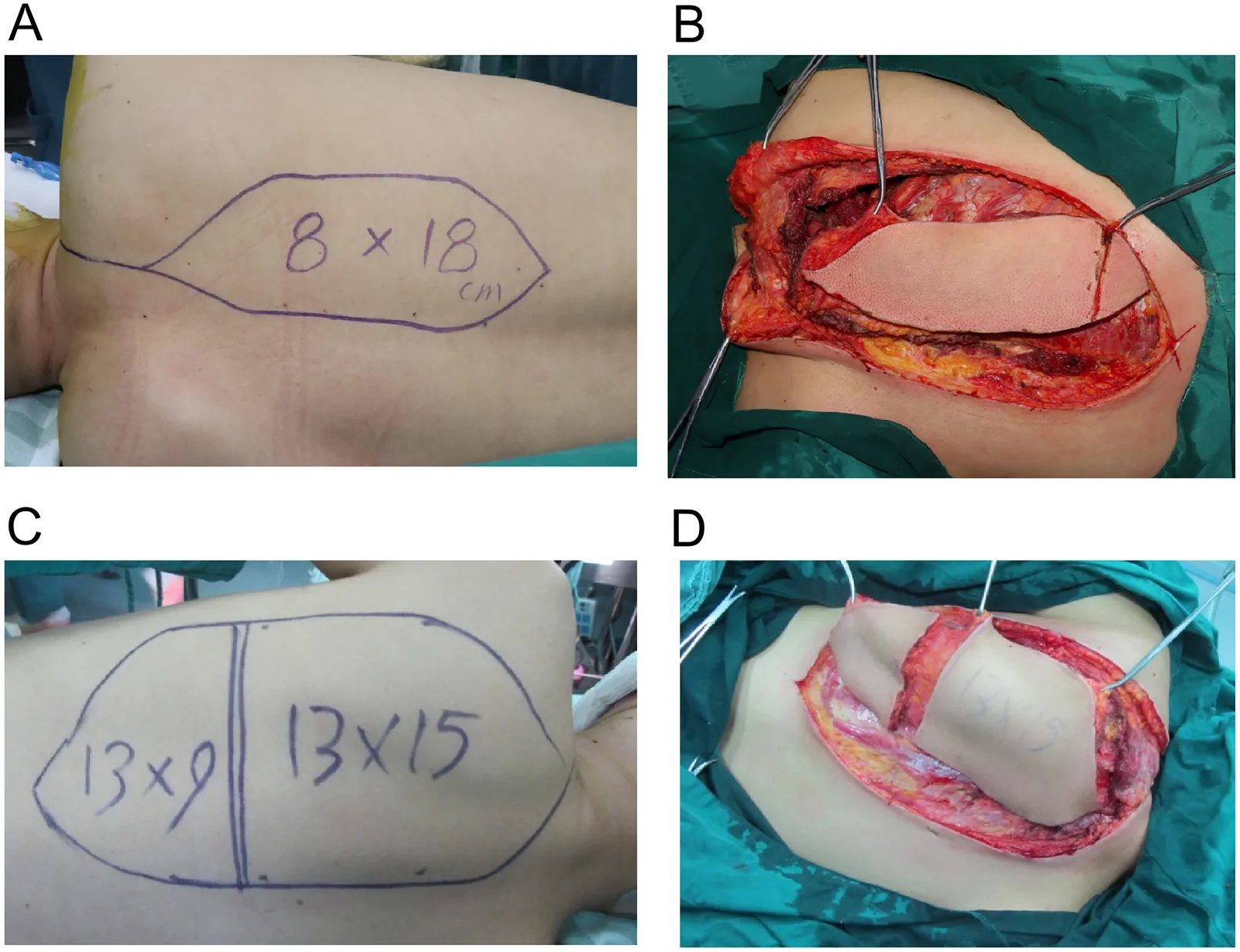
Harvest of the extended vertical lower trapezius island myocutaneous flap (eTMF). In Case 1, an 8 × 18 cm skin paddle was designed (A). A foldable skin flap was created with formation of the skin paddle and pedicle, while preserving the pedicle and the upper portion of the trapezius muscle (B). In Case 2, a foldable eTMF measuring 13.0 × (15 + 9) cm was designed (C). A foldable skin flap was created by excising a 2.5-cm-wide skin strip while preserving the pedicle and the upper portion of the trapezius muscle (D).

First, an eTMF was harvested under general anesthesia with tracheotomy. The surgical technique has been described in detail in our previous studies.^16^, ^17^, ^18^, ^19^ In this study, two foldable eTMFs were designed. For Case 1, an 8 cm × (13 + 5) cm skin paddle was used, and for Case 2, a 13.0 cm × (15 + 9) cm skin paddle was used (Fig. 4). In Cases 1 and 2, intraoperative blood loss during flap elevation was 90 mL and 110 mL, respectively. The duration of flap elevation was 90 min in Case 1 and 105 min in Case 2.

**Fig. 5:**
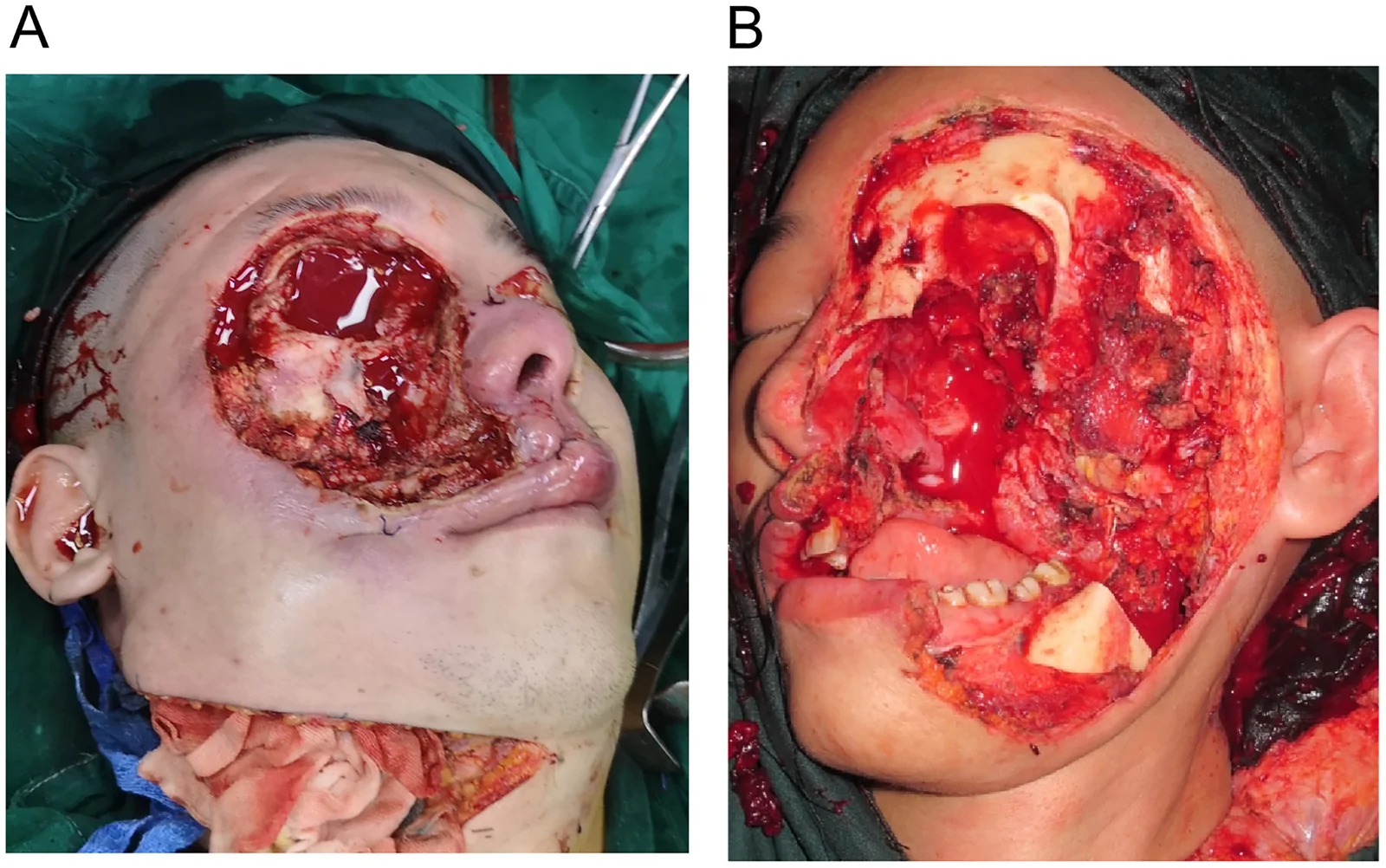
Patient 1 with AVMs involving the orbital region underwent en bloc resection and developed a maxillofacial defect (A). Patient 2 with AVMs and T4a squamous cell carcinoma of the left maxilla underwent en bloc resection combined with radical neck lymph node dissection, resulting in craniofacial defects (B).

Second, the same surgical team performed en bloc tumor resection after harvesting the eTMF. In Case 1, preoperative autologous donation of 4 units of red blood cells (RBCs) was performed. The right external carotid artery was ligated. An incision was made through normal skin at the tumor margin, and the tumor was dissected from the deep tissues. Hemostasis was achieved gradually using a Harmonic ACE+7 scalpel in advanced hemostasis mode, with additional vascular ligation as required. The resection included the right upper and lower eyelids, orbital contents, orbital floor, lacrimal gland, lacrimal sac, pterygopalatine fossa, maxilla, and full thickness of the cheek (Fig. 5A). In Case 2, preoperative autologous donation of 3 units of RBCs was performed. En bloc resection included both soft- and hard-tissue components. During soft-tissue resection, a Harmonic ACE+7 scalpel was used, with vascular ligation and suturing of larger muscle segments as necessary. Resection encompassed the left eyelids, orbital contents, partial nasal structures, full-thickness cheek, upper and lower lips, and bilateral neck lymph node dissection. Hard-tissue resection was performed using electric saws, and bone surfaces were sealed with bone wax to achieve hemostasis. The zygoma, maxilla, and mandible were removed (Fig. 5B). In Cases 1 and 2, intraoperative blood loss during en bloc tumor resection was 800 and 1200 mL, respectively.

**Fig. 6:**
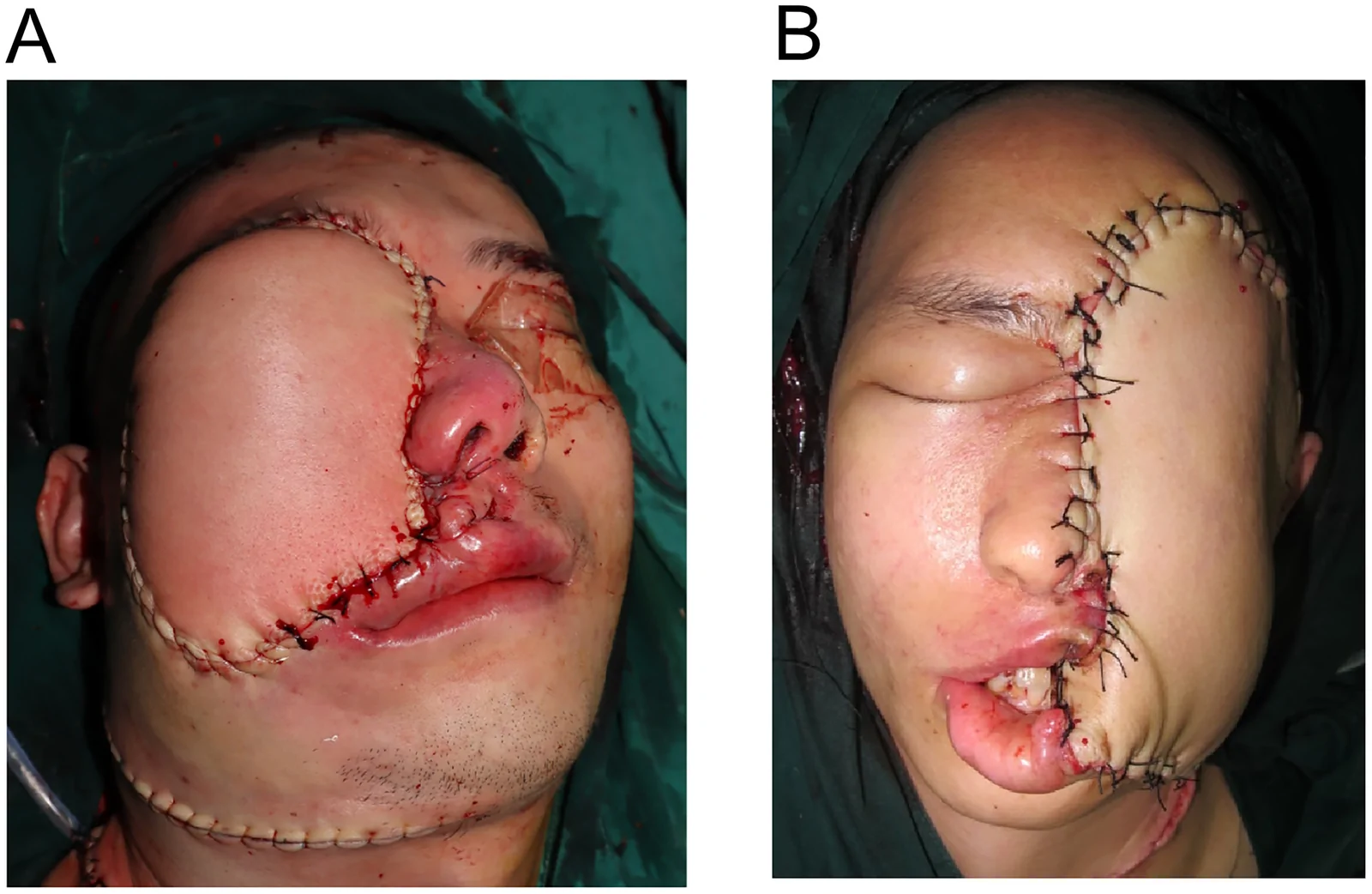
The folded skin flap provides both inner and outer lining for reconstruction of extensive head and neck defects. Frontal and lateral views of Case 1 (A), and frontal and lateral views of Case 2 (B), are shown.

Finally, the foldable eTMF was transferred to the head and neck region. The distal portion of the flap was turned to reconstruct the intraoral lining, and the medial portion was used to reconstruct the external lining of the head and neck. Next, the flap was secured in the appropriate anatomical position with sutures (Fig. 6).

## Results

**Fig. 7:**
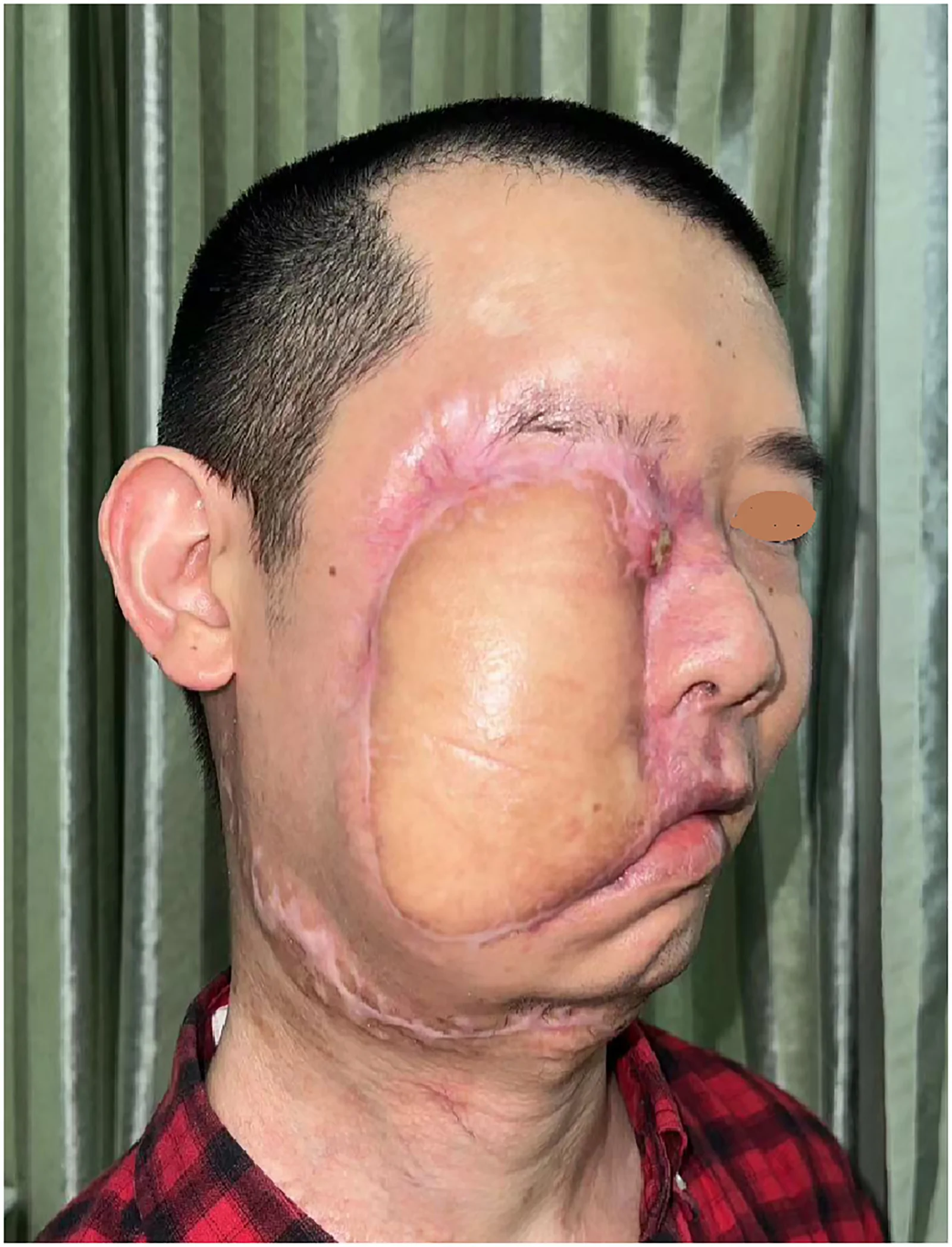
Patient 1 showed no evidence of disease at 36 months after surgery.

Both flaps survived, and no major complications occurred. All donor sites were closed, primarily without wound-healing problems. Postoperative head and neck appearance was satisfactory or acceptable in both patients. Oral intake was resumed gradually, and normal speech was ultimately restored. At 6 months after surgery, donor-site evaluation exhibited minimal posterior contour deformity, with mild restriction in upper-limb range of motion. The UW-QOL questionnaire results indicated that swallowing, chewing, and speech function had returned to preoperative levels. Overall quality-of-life scores improved by approximately 40% points, reaching 80% in Case 1 and 60% in Case 2. After 36 months of follow-up, Patient 1 showed no evidence of disease (Fig. 7). Patient 2 died of local recurrence 8 months after surgery, after declining adjuvant radiotherapy and chemotherapy (Table 2).

**Table 2:** Outcomes following the use of the eTIMF for reconstruction after resection in two patients with AVMs.

Outcomes	Patient 1	Patient 2
Tracheotomy / external carotid artery ligation	Yes / Yes	Yes / No
eTIMF size (cm)	8 × (14 + 4)	13.0 × (15 + 9)
Blood loss (mL)		
Flap elevation + tumor resection	90 + 800	100 + 1200
Red blood cell transfusion (units) autologous + homologous	4 + 0	3 + 3
Operative duration (min) Flap elevation + tumor resection	90 + 110	105 + 130
Tracheotomy cannula removal (day)	3	7
Speaking / oral feeding (day)	3/4	7/10
Head and neck appearance	Satisfactory	Acceptable
Donor-site deformity (back region)	Minimal	Minimal
Range of motion of the upper limbs	Mild restriction	Mild restriction
UW-QOL score (before / after surgery)		
Swallowing,	100 / 100	75 / 75
Chewing	100 / 100	75 / 75
Speech	100 / 100	75 / 75
Quality of life	40 / 80	20 / 60
Follow-up (months)	36	8
Status at last follow-up	No evidence of disease	Died of disease

AVM, arteriovenous malformations, UW-QOL: University of Washington Quality of Life Questionnaire; eTIMF, extended trapezius island myocutaneous flap.

## Discussion

We report two cases of life-threatening giant extracranial AVMs classified as Schobinger stage III/Yakes type IV involving the orbital region; Case 2 was additionally complicated by clinical stage T4a SCC of the left maxilla. According to the MDT-determined treatment strategy, the lesions were excised successfully en bloc, with radical neck dissection performed in Case 2. Two foldable eTMFs, measuring 8 cm × (14 + 4) cm and 13.0 cm × (15 + 9) cm, were used successfully to reconstruct extensive head and neck defects, including full-thickness cheek defects.

During surgery, meticulous vascular ligation and suturing of large tissue segments were performed. The Harmonic ACE+7 scalpel was used for eTMF harvesting and tumor resection, facilitating the sealing of small vessels, substantially reducing intraoperative bleeding, and achieving effective hemostasis. Total blood loss associated with flap elevation and tumor resection was 890 mL in Case 1 and 1300 mL in Case 2. In Case 1, transfusion was limited to 4 units of autologous RBCs. In Case 2, because of the more extensive surgical scope, transfusion included 3 units of autologous RBCs and an additional 3 units of allogeneic RBCs. The overall operative duration, including flap elevation and en bloc tumor resection, was relatively short, at 200 min and 235 min for Cases 1 and 2, respectively. Shortening the operative time is advantageous in reducing intraoperative blood loss and perioperative complications and may contribute to more rapid postoperative recovery.

Our study demonstrates that postoperative appearance after head and neck tumor resection and reconstruction was acceptable, with only mild posterior donor-site contour deformity and limited range of motion of the left upper limb. Swallowing, chewing, and speech function were restored to preoperative levels, and overall quality of life improved substantially. Patient 1 remained free of disease for 3 years after surgery. In contrast, Patient 2 did not receive adjuvant radiotherapy or chemotherapy and died of local recurrence 8 months after surgery.

These findings indicate that the MDT-determined therapeutic strategy is feasible. En bloc resection or radical surgery represents an effective treatment option for patients with life-threatening extracranial AVMs classified as Schobinger stage III/Yakes type IV involving the orbital region, even when accompanied by T4a SCC of the maxilla. The foldable eTMF is a reliable, large-volume flap for reconstruction of extensive head and neck defects, including full-thickness cheek defects.

The two cases of head and neck AVMs reported in our study were extremely challenging to manage and had disease durations exceeding 30 years. Despite multiple interventional treatments, including endovascular techniques, disease control was not achieved until the lesions progressed to a life-threatening stage and, in one case, underwent malignant transformation. Previous studies indicate that up to 80% of AVMs recur after embolization or resection. Incomplete resection or embolization may promote aggressive growth of residual lesions, with progression rates of up to 50% within the first 5 years and the potential for recurrence even after 10 years.^2^ The success of selective embolization is highly dependent on local vascular anatomy. The embolization efficacy for Yakes type IV lesions is inferior to that for Yakes types II and III, and type IV lesions generally require more extensive treatment.^6^ Proximal arterial ligation or embolization is not recommended, as lesions may develop collateral arterial supply, complicating subsequent endovascular management and precluding further embolization. Recent studies support a strategy of maximal safe embolization followed by direct or short-interval surgical total resection.^2^ Therefore, long-term postoperative follow-up is essential for early detection of recurrence. The adoption of an MDT approach is critical to guide treatment decisions, optimize therapeutic strategies, and achieve favorable clinical outcomes.

Extracranial AVM associated with malignant tumors or malignant transformation is rare. Previous studies have described radiation-induced malignant tumors following radiotherapy for intracranial AVMs, with a reported maximum incidence of 0.24%; glioblastoma is the most frequently observed histopathologic subtype.^20^, ^21^ Chen et al.^22^ reported two pediatric cases of malignant tumors of the upper lip—fibrosarcoma and rhabdomyosarcoma—that were initially misdiagnosed as hemangiomas. In our study, Case 2 involved an extracranial AVM concomitant with primary T4a SCC of the maxilla, the etiology of which remains unclear, and the therapeutic outcome was unfavorable. When extracranial AVMs are accompanied by malignant lesions, adjuvant radiotherapy and chemotherapy may be required as part of comprehensive oncologic management.

## Conclusions

Extracranial AVMs of the head and neck are progressive and invasive conditions that may result in life-threatening hemorrhage or coexist with malignant lesions. A therapeutic strategy determined by an MDT is feasible and effective. En bloc resection or radical surgery represents an appropriate treatment option for patients with life-threatening extracranial Schobinger stage III/Yakes type IV AVMs involving the orbital region, even when accompanied by T4a-stage SCC of the maxilla. The foldable eTMF is a large, highly reliable skin flap for reconstruction of extensive head and neck defects, including full-thickness cheek defects. As the report only includes two patients, this study is exploratory and further related research is necessary.

## Authors’ contributions

**Wei-liang Chen** conceived the study, oversaw the design of the study, oversaw all clinical and technical aspects of the study, helped analyze the data, provided guidance in the initial draft of the manuscript, and edited and approved the final version of the manuscript in its current form. **Bin Zhou** helped design the study, collected patient data, analyzed the results, wrote the initial draft of the manuscript, and edited and approved the final version of the manuscript in its current form. **Zi-xian Huang** and **Hai-gang Li** assisted in study design, collected patient data, and edited and approved the final version of the manuscript in its current form. **Rui Chen** and **You-yuan Wang** assisted in study design, collected patient data, and edited and approved the final version of the manuscript in its current form.All authors read and approved the final manuscript.

## Ethics approval and consent to participate

This study was approved by the university’s Institutional Review Board .

## Consent for publication

Informed written consent was obtained from each participant involved in the study.

## Patient consent for photo publication

Two patients received detailed oral and written information about the use of their pictures performed during pre- and post-operative follow-up. They signed an informed consent form before any study procedures, in which they authorized the picture's use for academic and scientific purposes.

## Funding

This work was supported by grants from 10.13039/501100001809National Natural Science Foundation of China (81772888 to Wei-liang Chen, 81702695 to Bin Zhou).

## Competing interests

The authors declare that they have no competing interests.

## Data Availability

Data sharing is not applicable to this article as no datasets were generated or analysed during the current study.
